# Pulse wave Doppler ultrasound of testicular arteries and their relationship with semen characteristics in healthy bulls

**DOI:** 10.1186/s40104-017-0229-6

**Published:** 2018-02-06

**Authors:** Alessia Gloria, Augusto Carluccio, Laura Wegher, Domenico Robbe, Claudio Valorz, Alberto Contri

**Affiliations:** 1 Faculty of Veterinary Medicine, University of Teramo, Località Piano D’Accio, 64100 Teramo, Italy; 2 Provincial Breeders Federation of Trento, Via delle Bettine 40, 38121 Trento, Italy

**Keywords:** Bull, Pulse wave Doppler, Sperm morphology, Testis, Ultrasound

## Abstract

**Background:**

Semen evaluation is used to estimate the testicular function. In bulls, the spermatozoa present in the ejaculate are the result of a process that begun more than 2 mo earlier, bequeathing a delayed depiction of the actual function of the testis. Since testis vascularization might be critical for the gonad function, selected pulse wave Doppler ultrasound parameters were assessed in this study, for instance the peak systolic velocity, the end diastolic velocity and the resistive index of the testicular artery along the spermatic cord, the marginal portion of the testicular artery and the intratesticular branches of the testicular artery both in healthy adult and young bulls. Correlations between these parameters and characteristics of semen that was collected numerous times, before and after the Doppler ultrasound examination.

**Results:**

The peak systolic velocity and the end diastolic velocity measured in the testicular artery along the spermatic cord (supratesticular artery – SA) were variable among the bulls and within individual bulls, likely due to the convoluted course of the vessel. The resistive index was found highly repeatable in the same bull. A reduction in the resistive index was found between the supratesticular artery and the marginal portion of the testicular artery (*P* < 0.01), and between the marginal portion of the testicular artery and the intratesticular branches of the testicular artery (*P* < 0.05). No differences were recorded for the pulse wave Doppler ultrasound parameters in young bulls compared with adults. A significant correlation was found between the resistive index of the marginal portion of the testicular artery and total sperm in the ejaculate (*r* = 0.516, *P* < 0.05), the immature sperm (*r* = 0.462, *P* < 0.05), the teratoid sperm (*r* = 0.375, *P* < 0.05), and the “Dag defect” sperm (*r* = 0.389, *P* < 0.05). Similarly, the resistive index of the intratesticular branches of the testicular artery were found correlated with the total sperm number in the ejaculate (*r* = 0.568, *P* < 0.05), the immature sperm (*r* = 0.523, *P* < 0.05), the teratoid sperm (*r* = 0.418, *P* < 0.05), and the “Dag defect” sperm (*r* = 0.341, *P* < 0.05).

**Conclusions:**

The data presented in this study suggest that the resistive index, measured at the marginal portion of the testicular artery, could be an easy-to-perform parameter to evaluate the spermatogenesis quality in young bulls and normal adults.

## Background

In the breeding soundness evaluation of the bull, the scrotal circumference and semen analysis are widely used as reference to assess the testicular function [1]. However, since the duration of spermatogenesis in the bulls takes approximately 62 d, and the epididymal transit takes 11 d [2], the mature spermatozoa that are present in the ejaculate had entered spermatogenesis approximately 70 d earlier. For this reason, the results of semen evaluation performed at any given time, reflect an actual testicular function with a significant delay [3]. This could affect our ability to monitor a progression of testicular diseases or to assess the effectiveness of treatment.

Testicular parenchyma is supplied by a long testicular artery, showing low oxygen tension [4–6] and low arterial capillary pressure, which was found to be only marginally higher than in venous counterpart [4, 7]. Furthermore, an alteration in the blood flow would result in a gonadal dysfunction [6]. Some studies performed in rats [8, 9] and in rams [10] have shown that long-term total ligation of the testicular artery or partial lumen occlusion caused arteriosclerosis in the testicular artery and selective damage to spermatogonia and preleptotene spermatocytes. Kay et al. [11] reported that a totally absent spermatogenesis, or existing only in a small proportion of seminiferous tubules, was obtained by the experimentally-induced restriction of the testicular artery in bulls, demonstrating the central role of the perfusion in the testicular function.

The ultrasonographic evaluation of the scrotal content represents one of the central investigations that can be exploited to assess the testicular function. Several studies tested the use of ultrasonography (US) to determine the normal testicular function [12] or to detect pathologies [13, 14]. The US evaluation with pixel intensity in the B-mode was found effective in monitoring the testicular maturation in the beef bulls [15, 16] or to evaluate the acute testicular damage after insulation [17], but had limited diagnostic value in absence of focal lesions of the testis in adult bulls [16].

High-resolution color Doppler US was recently proposed as a tool to evaluate the testicular vasculature and, in turn, the perfusion of the testis. In human medicine color Doppler US has been employed to evaluate testicular blood flow in pathological conditions [18, 19]. Furthermore, the quantitative evaluation of testicular blood flow by pulse wave Doppler ultrasound (PWDU) in human was suggested to predict the testicular function [20, 21]. In veterinary medicine, some studies have described the method for the PWDU in the dog [22, 23] and the horse [24–26], but the applicability of these techniques to evaluate the sperm production and testicular activity received limited attention. Additionally, the repeatability of the method carried out in these studies was not tested by a specific statistical design.

There are no data concerning the application of the PWDU to evaluate the testicular function in the bull. Consequently, the aim of the present study was to assess the associations between the selected PWDU parameters (peak systolic velocity – PSV; end diastolic velocity – EDV; resistive index – RI) obtained from the testicular artery and its branches, and sperm characteristics. The ultimate goal of the study was to verify if these PWDU parameters could be used to evaluate the actual status of the testis in the bull.

## Methods

### Animals

This study was performed on 18 Swiss Brown bulls, nine adult (mean 6.8 ± 2.3 yr, 4 to 7 yr) and nine young (mean 21.3 ± 3.6 mo, 17 to 24 mo) regularly used in artificial insemination (AI) programs. The bulls, owned by the Superbrown Consortium, were housed in the Alpenseme AI Center of the Provincial Breeders Federation of Trento (Ton, Trento, Italy). The animals were maintained according to the Italian legislation on animal care (DL No. 116, 27/01/1992). The owner of the animals gave his consent to the performed procedure. Aliquots for semen evaluations were part of the ejaculates routinely collected in the artificial insemination centre, and no semen collections were performed specifically for the study.

### Pulse wave Doppler ultrasound examination

For the ultrasound examination, the bull was restrained in a bovine individual steel stanchion, and a posterior approach to the scrotal region was used, without the need of sedative administration. The scrotal skin was cleaned, and 70% ethyl alcohol and ultrasonographic gel (Aquasonic 100, Parker Laboratories Inc., Fairfield, NJ, USA) were applied to increase the quality of the ultrasound image. A portable ultrasound machine Logiq Book XP (GE Healthcare, Wauwatosa, WI, USA) equipped with a 10-MHz linear probe (I739RS - GE Healthcare) was used. The scan depth was 10 cm for all the examinations.

The images were acquired at 10 MHz, and the sample volume was set to 1 mm. All the ultrasound machine settings remained unchanged during the trial. Initially, the probe was applied on the spermatic cord with horizontal orientation to examine the SA. Subsequently, the probe was moved to the rear face of the scrotum with vertical orientation and moved equatorially with the same orientation around the scrotum, in order to identify and examine the MA and the IA. After the vessel detection, the orientation of the probe was adjusted to achieve the visualization of at least 3-mm recognizable longitudinal section, the sample volume marker was pointed on the vessel, the angle of insonation was adjusted in the window below 60 degrees to obtain a valid velocimetric measures along the course of the artery, and at least three spectral curves were followed.

The peak systolic velocity (PSV, m/s), the end diastolic velocity (EDV; m/s), and the resistive index [RI; calculated as (PSV-EDV)/PSV] were measured by the internal algorithm. All the measurement procedures were repeated three times.

### Semen collection and evaluation

For each bull, semen collections and evaluations were performed d − 60 and d − 30 before the PWDU, the day of the PWDU (d 0), and d 30, d 60, and d 90 after the PWDU. Semen was collected using an artificial vagina in a sterile 13-mL plastic tube. The volume (VOL, mL) was estimated by the ejaculate weight measured with a precision balance CP6201 (Sartorius AG, Gottingen, Germany). The concentration (CONC, × 10^6^ sperm/mL) was estimated using Accucell photometer (IMV Technologie, L’Aigle, France). Total sperm/ejaculate (TSE, × 10^9^ sperm/mL) was calculated as: VOL × CONC.

#### Sperm membrane integrity evaluation

Membrane integrity (MI) was evaluated using a conventional epifluorescent double stain with propidium iodide (PI) and SYBR-14 fluorescent stain (LIVE/DEAD® Sperm Viability Kit; Molecular Probes Inc., Eugene, OR, USA) as previously described [27] with some modifications [28]. Briefly, an aliquot (1 mL) of diluted semen was incubated with 2.4 μmol/L of PI and 20 nmol/L of SYBR-14 at 37 °C under lightproof conditions. After 10 min, spermatozoa were immobilized with 10 μL of 3% glutaraldehyde, and 6 μL of this solution was placed on a slide. A cover slip was applied, and the stained spermatozoa were examined with an Olympus BX51 epifluorescence microscope (Olympus Italy, Milan, Italy). Spermatozoa with bright green fluorescence (SYBR-14) were considered viable, those partially or totally red (PI) were considered dead. The percentage of spermatozoa with membrane integrity was calculated on at least 400 spermatozoa for each sample*.*

#### Sperm morphology evaluation

Sperm morphology was evaluated using phase contrast microscopy (magnification: × 1000) after fixation with 0.9% NaCl solution with 3% glutaraldehyde [29]. In this study, immature (underdeveloped) sperm, double forms, acrosome defects (knobbed head or head with craters), decapitated sperm, teratoid head, diadem defect, pear-shape defect, macrocephalic head, microcephalic head, corkscrew defect, tail stumps, Dag defect, proximal droplets were considered major defects. Bent or kinked midpiece, distally coiled tail, bent tail, and distal cytoplasmic droplet were considered minor sperm defects [30]. Percentages of sperm abnormalities were calculated over evaluation of at least 200 spermatozoa.

#### Sperm motility evaluation

The sperm kinetic parameters were determined using a computer assisted sperm analyzer (CASA) IVOS 12.3 (Hamilton-Thorne Bioscience, Beverly, MA, USA). Motility parameters were collected and recorded by the analysis of 12 nonconsecutive fields. The anticollision algorithm was activated. Makler chamber was used for analysis [31]. The parameters considered in this study were total motility (TM, %), progressive motility (PM, %), average path velocity (VAP, μm/s), straight line velocity (VSL, μm/s), curvilinear velocity (VCL, μm/s), amplitude of lateral head displacement (ALH, μm), beat cross frequency (BCF, Hz), straightness (STR, as VSL/VAP, %), and linearity (LIN, as VSL/VCL, %). The software was set as previously reported [28, 32]. Spermatozoa with a VAP ≥ 80 μm/s and STR ≥ 75% were considered progressive.

### Statistical analysis

The data presented in this study were reported as mean ± standard deviation (SD). The data recorded for normality were tested using the Kolmogorov-Smirnov test. Since the data were normally distributed (*P* > 0.05), the PWDU parameters (PSV, EDV and RI), measured in triplicate, were analyzed using generalized linear model (GLM) for repeated measures while the testicle (right vs. left), the age (young vs. adult), and the type of the vessel (SA, MA, and IA) were considered fixed factors. As per the vessel type, the post-hoc evaluation was performed using the Scheffé test. For semen characteristics, VOL, CONC, TSE, MI, sperm abnormalities, and sperm kinematics, the GLM for repeated measures was used, considering the age of the bull as a fixed factor.

Possible correlations between the sperm parameters and the testicular vascular measurements were assessed by the calculation of the Pearson correlation coefficient.

The differences were considered significant when *P* ≤ 0.05. The statistical evaluations were performed using the SPSS 17.0 software (SPSS Inc., Chicago, IL, USA).

## Results

The PWDU of the testis was relatively simple in all bulls. Only the evaluation of the IA required more time for the measurement, due to the frequent movement of the bulls during the procedure. The pulse wave Doppler US parameters and the sperm characteristics were normally distributed (*P* > 0.05). All PWDU values (PSV, EDV, RI), were significantly higher (*P* < 0.01) in SA than in MA, which was in turn significantly higher (*P* < 0.05) than the IA for PSV, and RI. End diastolic velocity showed similar (*P* < 0.05) values in the MA and IA. No differences in the crude values were recorded in the corresponding region of the artery between young and adult bulls. In all cases, the PWDU values registered in the different regions of the testicular artery, were similar in the right and left testis (Table 1). The GLM for repeated measures showed that the values of PSV and EDV recorded in the SA artery were significantly variable (*P* < 0.01) in the same bull, but the MA and the IA were similar (*P* < 0.05). Resistive index measured in various regions of the testicular artery was similar within the same bull.

**Table 1:** Perfusion parameters measured in the spermatic cord testicular artery (SA), marginal portion of the testicular artery (MA), and intratesticular branches of the testicular artery (IA) of the bulls (*n* = 18)

		SA	MA	IA
		Mean ± SD	Mean ± SD	Mean ± SD
PSV, m/s	Right	14.1 ± 4.5^a^	6.3 ± 1.2^b^	5.7 ± 1.4^c^
	Left	13.8 ± 4.4^a^	6.5 ± 1.4^b^	5.8 ± 2^b^
EDV, m/s	Right	5.5 ± 2.3^a^	4.2 ± 1^b^	3.9 ± 0.9^b^
	Left	5.4 ± 1.9^a^	4.3 ± 0.9^b^	3.7 ± 0.8^b^
RI	Right	0.61 ± 0.1^a^	0.42 ± 0.08^b^	0.33 ± 0.08^c^
	Left	0.6 ± 0.1^a^	0.43 ± 0.09^b^	0.35 ± 0.08^c^

Peak systolic velocity (PSV, m/s); End diastolic velocity (EDV, m/s), resistive index (RI). Means bearing different superscripts (a and b) in a row differ significantly (*P* < 0.05)

Semen characteristics are summarized in Table 2. As revealed by the GLM for repeated measures, no significant changes in the semen quality were recorded along the study. The bull age significantly affected the volume, the concentration, and the total sperm number in the ejaculate (*P* < 0.01). On the other hand, sperm membrane integrity was similar in young and adult bulls. The total amount of sperm abnormalities ranged between 10 to 23% in all subjects, with mean values of 8.4 ± 3.2% in the young bulls and 14.3 ± 6.9% in the adults. However, the abnormal forms were highly variable among individuals (*P* < 0.01), as evidenced by the standard deviation (Table 3). No differences (*P* > 0.05) were found in the percentage of the different abnormalities between adult and young bulls. Furthermore, the percentage of total sperm abnormalities, such as the different type of sperm defects, were similar (*P* > 0.05) within samples collected from the same bull.

**Table 2:** Semen characteristics in young (*n* = 9) and adult (*n* = 9) bulls

	Yong bulls	Adult bulls
	Mean ± SD	Mean ± SD
Volume, mL	5.1 ± 1.5^a^	9.2 ± 2.3^b^
Concentration, ×10^6^/mL	856 ± 221^a^	1148 ± 324^b^
Total sperm per ejaculate, × 10^9^	5.6 ± 1.1^a^	9.1 ± 1.4^b^
Membrane integrity, %	87.2 ± 8.4^a^	86.8 ± 7.1^a^
Total motility, %	91 ± 4^a^	89 ± 5^a^
Progressive motility, %	84 ± 5^a^	81 ± 5^a^
VAP, μm/s	121.4 ± 30.6^a^	127.9 ± 32.7^a^
VSL, μm/s	108.9 ± 25.9^a^	99.3 ± 21.9^a^
VCL, μm/s	162.9 ± 53^a^	184.6 ± 43.6^a^
ALH, μm	5.6 ± 2.3^a^	6.8 ± 2.1^a^
BCF, Hz	38.1 ± 10.7^a^	40.2 ± 9.5^a^
STR, %	89 ± 9^a^	87 ± 7^a^
LIN, %	69 ± 7^a^	62 ± 6^a^

Average path velocity (VAP); straight line velocity (VSL); curvilinear velocity (VCL); amplitude of lateral head displacement (ALH); beat cross frequency (BCF); straightness (STR); linearity (LIN)

Means bearing different superscripts (a and b) in a row differ significantly (*P* < 0.05)

**Table 3:** Abnormal sperm morphology in young (*n* = 9) and adult (*n* = 9) bulls

	Young bulls	Adult bulls
	Mean ± SD	Mean ± SD
Major abnormalities, %	6.1 ± 2.31	10.1 ± 5.42
Immature sperm, %	0.4 ± 0.2	1.3 ± 0.7
Double forms, %	0.2 ± 0.2	0.3 ± 0.2
Acrosome defects (knobbed acrosome), %	0.5 ± 0.1	0.7 ± 0.3
Decapitated head, %	1.2 ± 0.3	2.1 ± 0.8
Teratoid heads, %	0.7 ± 0.2	1.1 ± 0.6
Diadem defects, %	0.1 ± 0.07	0.1 ± 0.1
Pear-shaped defects, %	0.2 ± 0.1	0.4 ± 0.2
Macrocephalic head, %	0.1 ± 0.1	0.1 ± 0.06
Microcephalic head, %	0.8 ± 0.2	1.1 ± 0.5
Corkscrew defects, %	0.1 ± 0.14	0.2 ± 0.16
Tail stumps, %	0.2 ± 0.1	0.2 ± 0.2
Strongly coiled tails, “Dag” defect, %	0.9 ± 0.3	1.4 ± 1.3
Proximal droplets, %	0.7 ± 0.3	1.1 ± 0.3
Minor abnormalities, %	2.3 ± 0.81	4.2 ± 1.28
Bent or kinked midpiece, %	0.3 ± 0.14	0.4 ± 0.16
Distal droplet, %	0.9 ± 0.3	1.4 ± 0.3
Bent tail, %	0.6 ± 0.2	1.3 ± 0.4
Coiled tail, %	0.4 ± 0.1	0.6 ± 0.1
Other abnormal cells, %	0.1 ± 0.07	0.5 ± 0.32

Significant correlations were found between the RI, measured in both the MA and the IA, and TSE (*r* = 0.516, *P* < 0.05; *r* = 0.568, *P* < 0.05, respectively), immature sperm (*r* = 0.462, *P* < 0.05; *r* = 0.523, *P* < 0.05, respectively), teratoid sperm (*r* = 0.375, *P* < 0.05; *r* = 0.418, *P* < 0.05, respectively), and sperm with “Dag defect” (*r* = 0.389, *P* < 0.05; *r* = 0.341, *P* < 0.05, respectively). The other seminal characteristics were not significantly correlated with the PWDU parameters.

## Discussion

The PWUD of the testicular vasculature was previously studied in several species, such as human [20, 21, 33–37], stallion [25, 38], dog [22, 23, 39–41], and rat [42], both in normal and pathological conditions. The PWDU of the testicular artery was suggested to be a useful procedure to evaluate the perfusion of the testis because the testis received the vascularization exclusively through this vessel. The testicular artery is a part of the spermatic cord, where the great coiling of the vessels disperses the heat reducing the working temperature of the testis [43]. Then, the unique marginal artery courses the length of the testis, under the corpus epididymis, to the ventral pole of the testis, where it branches in several small arterials that run in dorsal and lateral direction [44].

In our study, the RI recorded in the different locations of the testicular artery and its branches had similar values in different bulls, and within the same bull, while the PSV and the EDV measured in the SA were variable in the same bull. Our approach was different from those reported previously in other studies for stallions [25] and dogs [23], in which the repeated measures of the pulse wave Doppler parameters performed in the same vessel were averaged, and in turn the repeatability was not tested. The PSV and EDV variability in the SA could be the result of the convoluted course of the vessel in the testicular vascular cone. It is likely that the inability to follow the vessel with a reliable angle correction, such as the lack of a sufficient straight part to perform a clear measurement, led to a less accurate measurement of the parameter [20, 21]. As confirmed in our results, the EDV is known to be variable between measurements [45]. On the other hand, the RI appeared a more stable parameter for the evaluation of the blood supply to the testis, because of its independence from the angle of insonation [20].

Our data showed a progressive reduction in the PWDU parameters along the course of the testicular artery and its branches. The trend found in the present study in the bull confirmed the data reported in stallions [25] and dogs [22], although the absolute recorded values were different. This significant lessening in all parameters was not found in the dog, in which, on the contrary, the Doppler velocimetric parameters of the testicular artery measured in the spermatic cord were similar to those recorded in the marginal portion of the testicular artery [40]. In the bull, the parameters recorded in the MA were significantly higher than the values recorded in the IA. The values recorded in the IA were consistent with the data reported in a previous study on the fertile male dog [41]. A similar decrease has been previously reported in a study in humans [21].

As previously reported in stallions [25] and dogs [41], the bulls PWDU parameters were similar in the right testis when compared with the left testis. Furthermore, in healthy bulls the age seemed not to affect the blood perfusion of the gonads, since the values recorded in the adult and in the young bulls were assessed to be similar. According to data collected during this current research, the age was not found to represent a relevant factor in men [46]. On the other hand, the EDV in the stallion was significantly greater and RI was significantly lower for the 11–15 years age group than for the 16–22 years age group [25]. In a previous study, the RI had lower values in prepubertal boys than in those pubertal or postpubertal, whose RI was comparable with adults values [47], suggesting that the definitive vascularization of the testis is related to the acquisition of the reproductive function, rather to the increasing age.

The blood perfusion of the testis seemed effectively measured by RI in the MA and in the IA. However, the measurement of the PWDU parameters in the IA could be tedious, time consuming, and is referred to a part of the testis. On the other hand, RI in the MA could be considered representative of the whole testicular perfusion. The relevance of RI in indirectly measuring the testicular function was supported by the evidence that a significant increase of this parameter in the MA was recorded in stallions whose the testicular dysfunction was induced pharmacologically [38]. The fresh ejaculated semen characteristics of all bulls included in this study were similar to those recorded previously in normal ejaculates from bulls of the same breed [28, 48], and within the normal range previously proposed in dairy cattle [49]. The lack of a significant variability between repeated semen collections in the same animal suggested that the semen characteristics were relatively stable over the study duration, and thus representative of the testicular function of the bull. Since our study considered only healthy bulls with seminal parameters within normal range, the applicability of the PWDU parameter to verify the testicular function should be confirmed in bulls whose testicular function is compromised. Classically, the evaluation of the testicular function of the bull always includes semen analysis [49]. All diseases localized in the testis (orchitis, hypoplasia, degeneration, hypogonadism, torsion, neoplasia) may cause a reduction in the quality of semen [50, 51]. However, the evaluation of the seminal material provide information on the spermatogenesis started about 61 d before [2, 52, 53]. Consequently, the seminologic framework represents an inadequate instrument to measure the actual testicular efficiency. This aspect was corroborated by the lack of correlation between ultrasound testicular grayscale and seminal parameters recorded contextually [16, 17]. However, the correlations became significant with sperm characteristics recorded 2 to 4 wk after the ultrasound evaluation in an acute testicular degenerative changes produced by scrotal insulation [17].

Among the perfusion parameters, only the RI measured in the MA and in IA was significantly correlated with the TSE, the percentage of immature sperm, the teratoid sperm, and the sperm with “Dag” defect, without differences between adult and young bulls. The correlation between all these parameters and the RI corroborates that the perfusion of the testis could be related, at least partially, to the efficient function of the testis in normal bulls. The TSE could be considered a non-invasive parameter to estimate the testicular function in bulls in which the semen is routinely collected [54]. Immature and teratoid sperm were correlated with a defective spermatogenesis process, even if a limited percentage of these abnormalities could be present in normal ejaculates from fertile males [55, 56]. Although the sperm with strongly coiled tail, commonly termed “Dag” defect, were hypothesized to have a genetic origin, they are also mentioned among the defects of the spermatogenesis/maturation of spermatozoa, and were reported in normal ejaculates below 4% [57]. Our data are in agreement with results reported recently in stallions, whose RI were negatively correlated with the total number of progressively motile and morphologically normal spermatozoa [38]. These data are in contrast with those reported in a previous study carried out in dogs [23], whose contextual evaluation of blood flow and semen parameters showed significant negative correlations between RI, and sperm motility or HOST+ sperm, but not with total sperm in the ejaculate nor with abnormal sperm. In a recent study on normal and infertile dogs, the RI was found negatively correlated to the contextual total motility [41].

## Conclusions

The data reported in the present study suggest that the normal testicular function, resulting in normal sperm production, is correlated to the perfusion of the testis. Pulse wave Doppler ultrasound measures and indices of the blood flow in the bull testis, especially RI obtained from the marginal part of the testicular artery, can be proposed as objective parameters of testicular function occurring at the same time as the PWDU evaluation is performed.

## Abbreviations

ALH: amplitude of lateral head displacement
BCF: beat cross frequency
CASA: computer assisted sperm analyzer
CONC: concentration
EDV: end diastolic velocity
GLM: general linear model
IA: intratesticular branches of the testicular artery
LIN: linearity
MA: marginal portion of the testicular artery
MI: membrane integrity
PI: propidium iodide
PM: progressive motility
PSV: peak systolic velocity
PWDU: pulse wave Doppler ultrasound
RI: resistive index
SA: spermatic cord testicular artery
SD: standard deviation
STR: straightness
TM: total motility
TSE: total sperm/ejaculate
US: ultrasound
VAP: average path velocity
VCL: curvilinear velocity
VOL: volume
VSL: straight line velocity
